# Immobilization of Murine Anti-BMP-2 Monoclonal Antibody on Various Biomaterials for Bone Tissue Engineering

**DOI:** 10.1155/2014/940860

**Published:** 2014-07-23

**Authors:** Sahar Ansari, Marcelo O. Freire, Eun-Kyoung Pang, Alaa I. Abdelhamid, Mohammad Almohaimeed, Homayoun H. Zadeh

**Affiliations:** ^1^Laboratory of Immune Regulation and Tissue Engineering, Herman Ostrow School of Dentistry, University of Southern California, Los Angeles, CA, USA; ^2^Department of Applied Oral Sciences, The Forsyth Institute, Cambridge, USA; ^3^Department of Oral Medicine, Infection and Immunity, Harvard School of Dental Medicine, Boston, MA, USA; ^4^Department of Periodontology, Graduate School of Medicine, Ewha Womans University, Seoul, Republic of Korea; ^5^Dental Research Center (DRC) and Tissue Engineering Unit (TERU), Qassim College of Dentistry, Qassim University, Saudi Arabia; ^6^Laboratory of Immune Regulation and Tissue Engineering (LITE), Division of Periodontology, Diagnostic Sciences & Dental Hygiene, Ostrow School of Dentistry, University of Southern California, 925 34th Street, Room 4278, Los Angeles, CA 90089-0641, USA

## Abstract

Biomaterials are widely used as scaffolds for tissue engineering. We have developed a strategy for bone tissue engineering that entails application of immobilized anti-BMP-2 monoclonal antibodies (mAbs) to capture endogenous BMPs in vivo and promote antibody-mediated osseous regeneration (AMOR). The purpose of the current study was to compare the efficacy of immobilization of a specific murine anti-BMP-2 mAb on three different types of biomaterials and to evaluate their suitability as scaffolds for AMOR. Anti-BMP-2 mAb or isotype control mAb was immobilized on titanium (Ti) microbeads, alginate hydrogel, and ACS. The treated biomaterials were surgically implanted in rat critical-sized calvarial defects. After 8 weeks, *de novo* bone formation was assessed using micro-CT and histomorphometric analyses. Results showed *de novo* bone regeneration with all three scaffolds with immobilized anti-BMP-2 mAb, but not isotype control mAb. Ti microbeads showed the highest volume of bone regeneration, followed by ACS. Alginate showed the lowest volume of bone. Localization of BMP-2, -4, and -7 antigens was detected on all 3 scaffolds with immobilized anti-BMP-2 mAb implanted in calvarial defects. Altogether, these data suggested a potential mechanism for bone regeneration through entrapment of endogenous BMP-2, -4, and -7 proteins leading to bone formation using different types of scaffolds *via* AMOR.

## 1. Introduction

The goal of bone tissue engineering is the regeneration of a construct that matches the physical and biological properties of the natural bone tissue and reestablishes function [1]. Bone tissue reconstruction is usually necessary due to congenital anomalies, infection, trauma, and skeletal diseases. Autologous and allogenic bone grafts are currently the main treatment options and comprise about 90% of grafts performed each year [1, 2]. However, there are several disadvantages associated with these modalities of treatment. These include significant potential morbidity of the donor site, operative and recovery time, and high expense of autologous grafts harvesting. Moreover, osteoconductive graft materials such as allografts, xenografts, and alloplastic material have limited ability to repair large defects, due to their inherent inability to initiate bone formation. For these reasons, alternative bone regeneration treatment modalities are desirable. Bone tissue engineering strategies have offered promising alternatives, developing biological bone substitutes that restore, maintain, or improve bone tissue function [3]. Bone tissue engineering aims to combine biomaterial scaffolds, cells, and molecular signals that can mediate tissue regeneration, matching the physical and biological properties of the natural tissue [3–5].

Currently, there are multiple bone tissue engineering strategies available, including gene therapy, stem cell therapy, exogenous growth factors, or a combination of these strategies. Growth factors such as bone morphogenetic proteins (BMPs), platelet-derived growth factors (PDGFs), and insulin-like growth factors (IGFs) have been utilized for bone tissue engineering with promising results [6–8]. Several* in vitro* studies have confirmed that BMP-2, BMP-4, and BMP-7 have the ability to stimulate the differentiation of osteoprogenitor cells into mature osteoblasts. Preclinical and clinical studies have demonstrated the osteoinductive potential of some BMPs, leading to the FDA approval of recombinant human BMP-2 (rhBMP-2) and rhBMP-7 for clinical applications [9–12]. However, there are a number of limitations to the application of exogenous rhBMPs, including reduced potency compared to their endogenous counterparts, requiring the administration of superphysiologic concentrations which in turn leads to significant side effects and high cost [13, 14].

An alternative treatment option to the administration of exogenous rhBMP-2 is the application of anti-BMP-2 monoclonal antibodies (mAbs) immobilized on a solid scaffold, in an effort to capture endogenous BMP-2. This approach, termed antibody-mediated osseous regeneration (AMOR), was first reported by Freire et al. [15]. In previous studies, immobilized murine anti-BMP-2 mAbs were immobilized on absorbable collagen sponge (ACS) and implanted within rat calvarial defects, demonstrating repair of the bone defects [15]. The* in vivo* osteogenic action of AMOR was later characterized by increased endogenous BMP-2, BMP-4, and BMP-7 in the microenvironment of the defect [16]. Consistent with our hypothesis that the osteogenic mechanism of AMOR is due to the capture and biologic action of endogenous BMPs, the initial regulatory mechanism has been shown to be mediated by the Smad intracellular signaling pathway [17]. While these mechanisms have begun to elucidate the osteogenic actions of AMOR, it is unknown whether the use of more versatile biomaterials, such as titanium or alginate, influences bone regeneration mediated by anti-BMP-2 mAbs.

In view of the important role of biomaterials in bone regenerative therapies, it will be desirable to examine their role in AMOR [18, 19]. ACS has been a convenient scaffold in our previous studies [15–17] and has been approved by FDA as a carrier for rhBMP-2 [20]. Moreover, because of its radiolucent properties, it is simple to demonstrate* de novo* bone formation. While ACS has excellent biocompatibility and did not interfere with AMOR in previous studies, its mechanical properties and rapid resorption are considerable deficiencies. Hence, the goal of this study was to evaluate the relative merits of alternative scaffolds with varying chemical, physical, and mechanical properties, including titanium and alginate. The efficacy of three different biomaterials has been compared in the immobilization of anti-BMP-2 mAbs for AMOR.

## 2. Materials and Methods

### 2.1. Materials

3G7 mAb (Abnova, Taipei, Taiwan), a murine monoclonal anti-BMP-2 antibody, was used in this study. Isotype-matched mAb (Iso, anti-rabbit IgG mAb, Biovision, Mountain View, CA) with no specific affinity to BMP-2 was used as the negative control. Anti-BMP-2 and isotype control mAbs were diluted with plain phosphate-buffered saline (PBS) at 25 *μ*g/mL and immobilized on each of the scaffolds according to the protocol previously reported by Freire et al., 2011. Three different scaffold materials were used in this study, including grade IV titanium microbeads with 250 *μ*m diameter (Sybron Dental Implants, Orange, CA), alginate hydrogel (NovaMatrix FMC Biopolymer, Norway), and ACS (Helicote, Miltex, Plainsboro, NJ). The effect of alginate volume of the dilution of the mAb was considered.

### 2.2. *In Vitro* mAb Binding and Release Kinetics Study

In order to evaluate the kinetics of murine anti-BMP-2 mAb release from each scaffold, 25 *μ*g/mL of mAb was immobilized on each scaffold (titanium microbeads, alginate hydrogel, and ACS) according to methods already described in the literature [17]. The mAb-loaded scaffolds were suspended in 5 mL of PBS (pH = 7.4). At various time points (1, 3, 7, and 14 days), the amount of released mAb was determined by UV absorption spectroscopy (Beckman, Brea, CA). In addition, the retained mAb was detected with FITC-conjugated goat anti-mouse IgG antibody (Santa Cruz Biotechnology Inc., CA) and measured using confocal laser scanning microscopy (CLSM). The fluorescence intensity was quantified by Spot analysis software (SPOT Imaging Solutions, Sterling Heights, MI).

### 2.3. Rat Critical Size Calvarial Defect

Thirty 2-month-old virgin female Sprague-Dawley rats (Harlan Laboratories, Livermore, CA) were housed at 22°C under a 12 h light and 12 h dark cycle and fed* ad libitum* (Purina Inc., Baldwin Park, CA). All animals were treated according to the guidelines and regulations for the use and care of animals at the University of Southern California. Full-thickness skin flaps were raised, exposing the parietal bones. 7 mm diameter defects in the parietal bones were generated using a trephine under copious saline irrigation. Each of the scaffold materials containing 25 *μ*g/mL of mAbs was placed inside each of the calvarial defects. At the end of the treatment period, 8 weeks after implantation, animals were sacrificed in a CO_2_ chamber and the skulls were harvested and stored in buffered formalin until further analysis.

### 2.4. Micro-CT Analysis

Retrieved specimens from the animals were scanned using a high-resolution micro-CT system (MicroCAT II, Siemens Medical Solutions Molecular Imaging, Knoxville, TN) for evaluation of ectopic mineralization. The specimens were scanned at widths of every 10 *μ*m at 60 kV and 110 *μ*A at a resolution of 20 *μ*m. Bone volume fraction (BV/TV) for each construct was calculated.

### 2.5. Histochemical Analysis

For histochemical analysis, the retrieved specimens were fixed with 4% (v/v) paraformaldehyde for 30 min at room temperature and then placed in PBS for 15 minutes prior to dehydration. Serial dehydration was achieved by placing the specimens in a sequential series of increasing ethanol concentrations to remove all the water. The ethanol was then completely replaced with increasing concentrations of xylene solution followed by a 100% xylene step prior to incubation with paraffin-saturated xylene at room temperature overnight. The specimens were then serially sectioned (6 *μ*m) and adhered to glass slides. The paraffin was completely removed by immersion in xylene, followed by decreasing ethanol concentrations, and then by washing with tap water. The sections were stained with hematoxylin and eosin (H&E). Images were captured using an Olympus DP50 digital camera (Olympus Optical Co., Japan) and analyzed using Analysis imaging software (Soft Image System GmbH, Germany).

### 2.6. Scanning Electron Microscopy (SEM)

In order to characterize the morphology of the scaffold materials used in this study and the early interaction of the implanted scaffolds and cells, scanning electron microscopy (SEM) (JEOL 5300, Peabody, MA) was used. The specimens were harvested from the animals 24 hrs after implantation. They were then rinsed with 2 mL of PBS and fixed with 1% glutaraldehyde overnight. Samples were dehydrated using graded alcohol solutions and sputter-coated with gold.

### 2.7. Confocal Laser Scanning Microscopy (CLSM)

In order to show the capacity of the murine anti-BMP-2 mAb immobilized on different scaffolds to attract and hold BMP-2, -4, and -7 ligands, CLSM was utilized. Briefly, specimens were retrieved eight weeks after implantation, fixed in 10% formalin solution, dehydrated in an ascending series of ethanol solutions, and embedded in paraffin. Six-micrometer sections were cut using a microtome and mounted on glass slides. For immunofluorescence staining, deparaffinized samples were treated with 3% H_2_O_2_, followed by a blocking buffer (1% BSA and 0.25% Triton X-100 in PBS), stained with rabbit polyclonal anti-BMP-2, BMP-4, and BMP-7 antibodies (Abcam, Cambridge, MA) at 4°C overnight, and detected using Alexa Fluor-conjugated secondary antibody (1 : 200 dilution; Invitrogen) using CLSM (Fluoview FV10i, Olympus Corp., Tokyo, Japan). The fluorescence intensity was analyzed and quantified by Spot analysis software (SPOT Imaging Solutions, Sterling Heights, MI) with the same fluorescence threshold.

### 2.8. Statistical Analysis of Data

Quantitative data were expressed as mean ± standard deviation (SD). One-way and two-way analyses of variance (ANOVA), followed by Tukey's test at a significance level of *α* = 0.05, were used for the comparison of multiple sample means.

## 3. Results

### 3.1. *In Vitro* Binding and Release Characteristics of Anti-BMP-2 mAb

**Figure 1:**
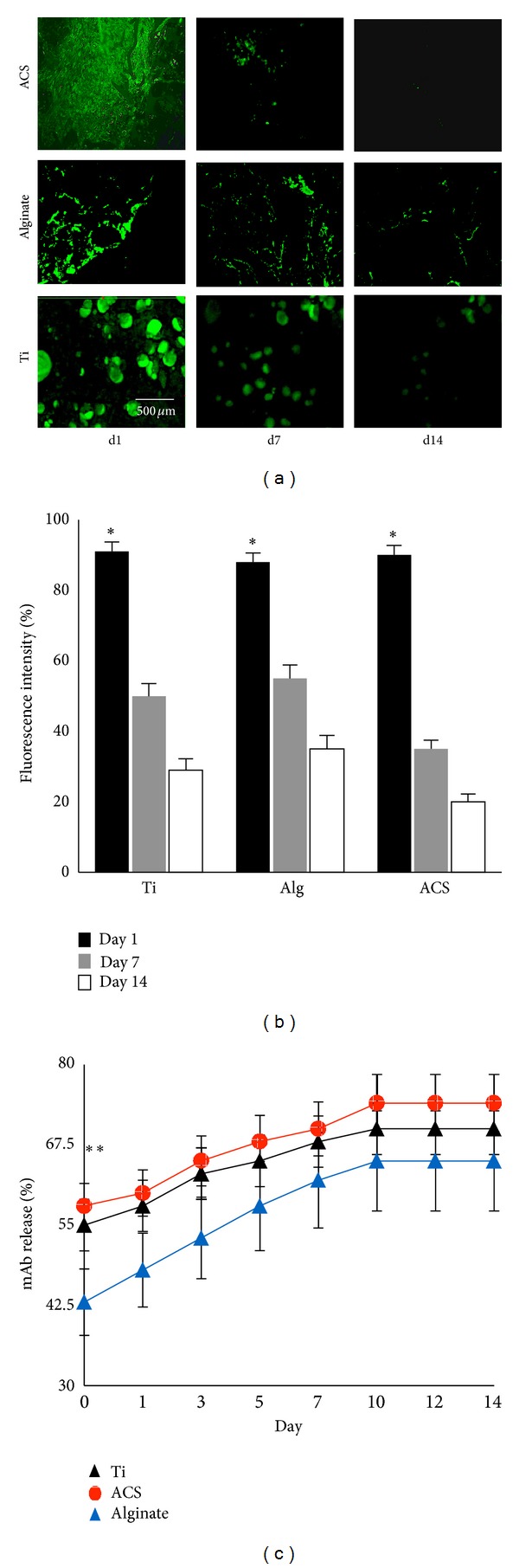
Characterizationof the* in vitro* binding and release profile of murine anti-BMP-2 mAb-loaded scaffolds. (a) CLSM analysis showing binding of anti-BMP-2 mAb on each scaffold detected by FITC-conjugated goat anti-mouse secondary antibody. Day 1 represents detection of binding of anti-BMP-2 mAb immediately after immobilization of the mAb on the scaffolds, confirming that murine mAb is retained on all tested scaffolds for up to two weeks* in vitro*. (b) Quantitative analysis of fluorescence intensity showing initial binding (day 1) of anti-BMP-2 mAb to the scaffolds and the* in vitro *persistence of anti-BMP-2 at 7 and 14 days later (*n* = 4). (c) The* in vitro* release of anti-BMP-2 mAb was calculated by measuring mAb concentrations in solution at various time points. *P < 0.05.

A study of* in vitro* binding and release kinetics was performed to examine potential differences in the binding and release profile of the murine mAb on the three different scaffolds. Results demonstrated that immediately after immobilization of anti-BMP-2 mAb, the levels of the antibody detected on all 3 scaffolds were equivalent (Figures 1(a) and 1(b)). Approximately 20% of mAb remained on the scaffolds after 2 weeks of* in vitro *incubation. The release profile of the murine mAb from each of the scaffolds showed sustained release for up to 14 days (Figure 1(c)). While alginate hydrogel showed a significantly lower (*P* < 0.05) initial release profile, no significant difference (*P* > 0.05) was observed in the amounts of release after day 3. Since ACS and alginate are bioresorbable scaffolds, we hypothesized that the kinetics of mAb retention and release were likely to be different* in vivo.*

### 3.2. *In Vivo* Bone Regeneration

**Figure 2:**
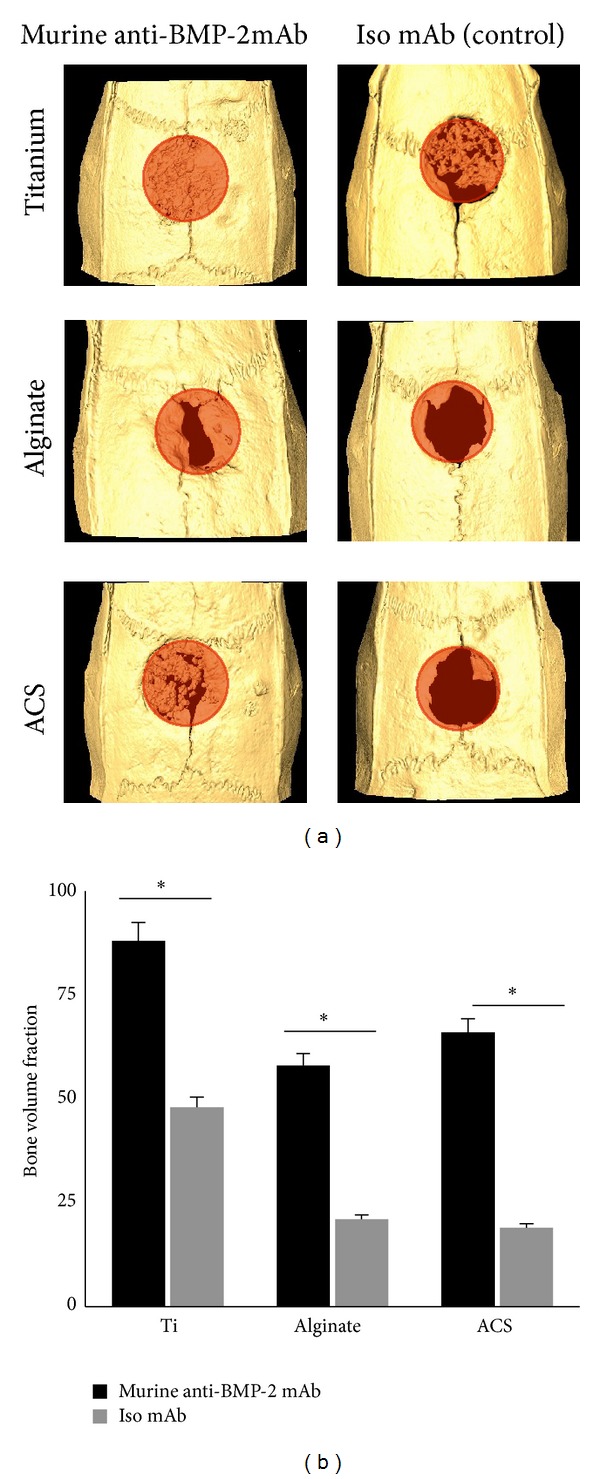
(a) Micro-CT images of rat calvarial defects 8 weeks after implantation of different biomaterials preloaded either with anti-BMP-2 mAb or isotype mAb as the negative control. (b) Quantitative analysis via micro-CT images showing the bone volume fraction (BV/TV) for each group (*N* = 4). *P < 0.05.

Micro-CT analysis (Figure 2(a)) showed a significant volume of* de novo* bone formation within the calvarial defects implanted with each of the three scaffolds immobilized with anti-BMP-2 mAb. In contrast, substitution of the mAb with isotype-matched control mAb did not mediate a significant degree of calvarial bone repair after 8 weeks of implantation. Quantified micro-CT results confirmed that sites with anti-BMP-2 mAb on Ti microbeads exhibited the largest volume of bone formation. However, it should be noted that Ti microbeads contributed to this large volume, as they are radiopaque and are not biodegradable. No significant difference was observed between the ACS and alginate groups (*P* > 0.05) (Figure 2(b)).

**Figure 3:**
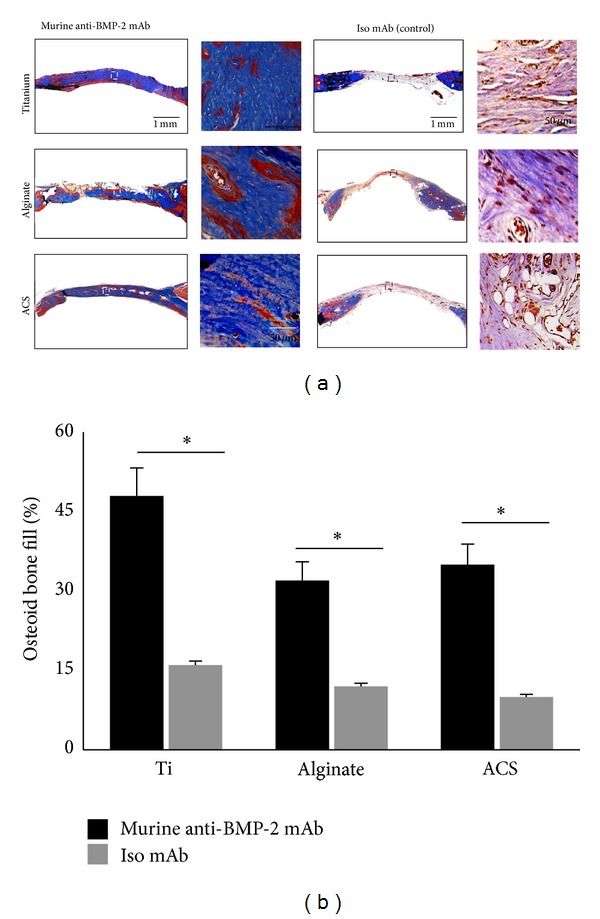
(a) Histological analysis of rat calvarial bone defects implanted with anti-BMP-2 mAb immobilized on scaffolds showing presence of vital bone in implantation sites. No evidence of bone formation was observed in sites implanted with isotype-matched control Ab. Collagen exhibited the most compression, followed by alginate, while titanium had the best tissue volume maintenance. (b) Histomorphometric analysis of rat calvarial bone defects implanted with anti-BMP-2 mAb immobilized on 3 different scaffolds. Histomorphometric analysis was performed on Trichrome-stained sections and percentage of new bone formation was quantified. No significant difference was observed between the proportions of new bone formation for each biomaterial (*N* = 4). *P < 0.05.

The histological analysis of rat calvarial defects implanted with anti-BMP-2 mAb immobilized on 3 different scaffolds is presented in Figure 3(a). The histomicrograms illustrated the presence of vital bone, indicated by the presence of osteocytes in lacunae within each of the scaffolds with immobilized anti-BMP-2 mAb. The degree of bone repair was significantly higher in sites with immobilized anti-BMP-2 mAb than in sites with isotype-matched control mAb. Due to their biodegradability, collagen scaffolds exhibited the most volumetric shrinkage, followed by alginate. Anti-BMP-2 mAb immobilized on titanium exhibited the largest volume of bone within the calvarial defects (*P* < 0.05). The histomorphometric analysis (Figure 3(b)) showed no significant difference between the proportions of* de novo* bone formation between alginate and ACS. Ti microbeads showed the largest amount of bone regeneration, followed by ACS. Alginate hydrogels samples showed the least amount of regenerated bone. The isotype mAb groups demonstrated significantly lower amounts of bone regeneration (*P* < 0.05). It is notable that the morphology of regenerated bone in the sites implanted anti-BMP-2 mAb and each of the three scaffolds was normal with no evidence of inflammation or any adverse effects.

### 3.3. SEM Analysis of Different Scaffolds

**Figure 4:**
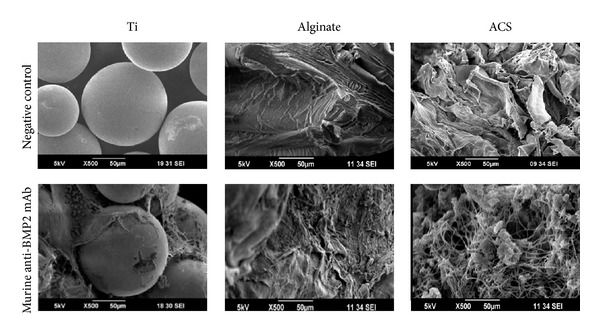
Representative SEM photomicrographs of scaffolds prior to implantation (-) or with immobilized murine anti-BMP-2 retrieved 24 hours after implantation into rat critical-sized calvarial defects. Significant cellular infiltration and adhesion were observed on scaffolds immobilized with murine anti-BMP-2 mAb. Both alginate and ACS scaffolds had porous structure while the spheroidal Ti microbeads had an average diameter of 250 *μ*m.

The morphology of the scaffold materials and the initial interaction of host tissues and cells with implanted scaffolds were characterized using SEM. The representative SEM photomicrographs of pristine scaffolds, as well as scaffolds with immobilized anti-BMP-2 following retrieval 24 hours after implantation into rat calvarial defects, are shown in Figure 4. The SEM images confirmed that both alginate and ACS scaffolds had porous structures, while the spheroidal Ti microbeads appeared to have relatively smooth surface. Significant cellular infiltration was observed on all the scaffolds immobilized with anti-BMP-2 mAb. Comparatively, scaffolds immobilized with isotype control mAb exhibited significantly less cellular infiltration (data not shown). The cells infiltrating anti-BMP-2 mAb-immobilized scaffolds appeared adherent with spreading on these scaffolds. Greater cell infiltration and adhesion were observed onto ACS and alginate hydrogel scaffolds.

### 3.4. CLSM Analysis

**Figure 5:**
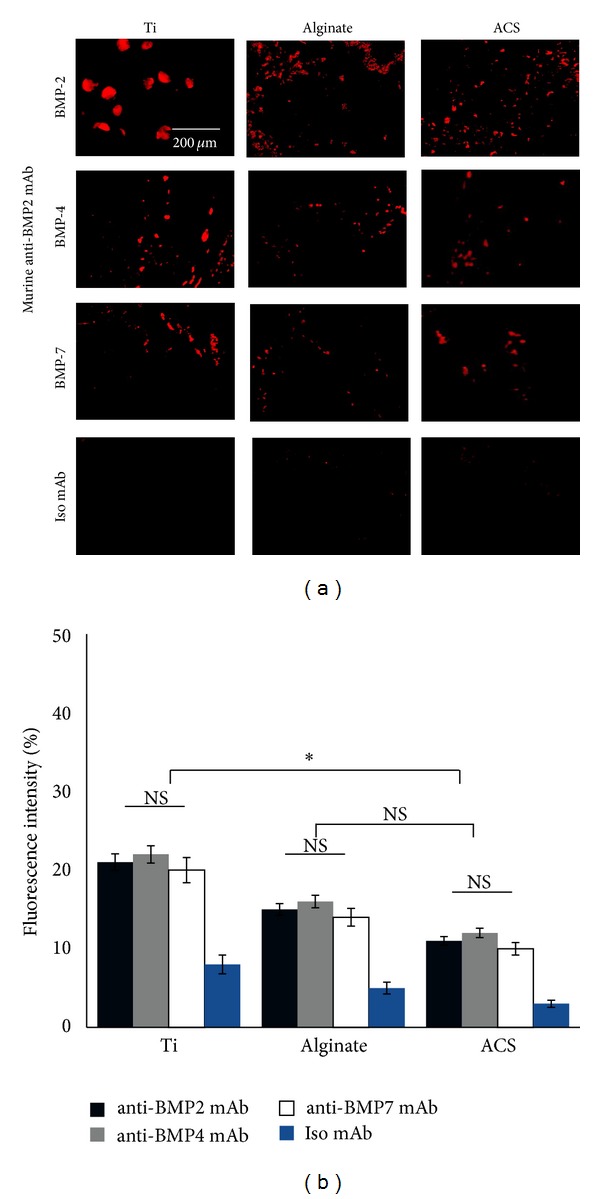
Localization of BMP-2, BMP-4, and BMP-7 antigens within defect sites with immobilized anti-BMP-2 mAb following* in vivo *implantation and retrieval after 8 weeks. (a) Representative CLSM images of titanium, alginate, and ACS groups with immobilized anti-BMP-2 mAb harvested from calvarial defects after 8 weeks. The immunofluorescence results revealed the capacity of the murine mAb to attract and hold BMP-2, -4, and -7 ligands. Scaffolds immobilized with nonspecific isotype mAb failed to show any positive staining. (b) Quantitative analysis of red fluorescence intensity of the images shown in (a). *N* = 4 for each group. *P < 0.05; NS: not significant.

In order to evaluate the capacity of the murine anti-BMP-2 mAb immobilized on different scaffolds to bind BMP-2, -4, and -7* in vivo*, the 3 scaffolds with immobilized anti-BMP-2 mAb were implanted in calvarial defects. The animals were sacrificed 8 weeks after implantation. CLSM analysis confirmed that murine anti-BMP-2 mAb immobilized on different scaffolds exhibited significant binding of BMP-2, BMP-4, and BMP-7 ligands following implantation (Figure 5(a)). As expected, the defects implanted with isotype-matched control mAb failed to bind BMP-2, -4, and -7 ligands. Results revealed the capacity of the murine mAb to localize increase concentrations of BMP-2, -4, and -7 ligands in all tested scaffolds. Titanium specimens showed higher fluorescence intensity (*P* < 0.05), while no significant difference was observed between the fluorescence intensity levels of alginate and ACS (*P* > 0.05) (Figure 5(b)).

## 4. Discussion

Implanted autogenous, allogeneic, xenogenic, and synthetic biomaterials are the common treatment modalities currently used for bone regeneration in craniofacial reconstructive surgeries and for other areas of regenerative medicine. While autologous grafts are considered the gold standard, they have many limitations; allografts, xenografts, and alloplastic biomaterials have therefore been used as alternatives. These biomaterials have found clinical applications in the reconstruction of large osseous defects. However, due to their lack of osteoinduction and unpredictable resorption rates, variable clinical outcomes have been observed. Recombinant BMPs have shown promise clinically as an alternative bone regeneration therapy [21–25]. BMP-2 is a member of the TGF-*β* family that assembles into a biologically active homodimer and binds to heterodimeric type I and type II receptors for BMP-2 [26, 27]. Other osteogenic BMPs include BMP-4 and BMP-7. Currently, the FDA has approved rhBMP-2 and rhBMP-7 for repair and regeneration of skeletal defects. However, there are several drawbacks to the application of recombinant growth factors, including their supraphysiologic dose requirement and some potentially serious side effects, as well as high cost. Our laboratory has therefore introduced AMOR as an alternative strategy to the current approaches of administering exogenous growth factors [15, 16].

It has been proposed that appropriate signaling molecules acting on progenitor cells within a suitable scaffold can lead to tissue regeneration. Our previous studies have established that when anti-BMP-2 mAbs are implanted* in vivo*, they can capture endogenous BMP-2, BMP-4, BMP-7 that provide the osteogenic signals to progenitor cells to regenerate bone [15, 16, 23]. Therefore, the current study sought to compare the efficacy of various scaffolds in the pursuit of optimizing this novel strategy. To that end, we examined the suitability of three different biomaterials with different physical and chemical properties as scaffolds when immobilized with anti-BMP-2 Abs for AMOR.

The results of the present study demonstrated that all of the tested biomaterials (Ti, alginate, and ACS) can be utilized as drug delivery vehicles for immobilized anti-BMP-2 mAb. Moreover, all three scaffolds have favorable binding and release profile characteristics. Alginate hydrogel showed a significantly lower initial release profile, with release characteristics becoming comparable to the other tested biomaterials after day 3. This phenomenon might be due to surface adsorption of the murine mAb on Ti and ACS, while the mAb was encapsulated within the alginate hydrogel. Due to the biodegradability of ACS and alginate, it is likely that the kinetics of mAb anti-BMP-2 retention and release will be different* in vivo.*

We also confirmed that all three scaffolds, when functionalized with the murine anti-BMP-2 mAb, mediated bone regeneration within calvarial defects. Several differences in the outcomes were noted, which could affect their potential clinical applications. Both ACS and alginate are biodegradable materials and, as such, their volumes decreased after implantation. In contrast, titanium is a biologically stable material and maintained its volume. Titanium is used extensively in orthopedic and dental implant therapies, and anti-BMP-2 mAb could potentially be utilized as a surface modification strategy in such applications. It may be possible to exploit the modulatory effects of anti-BMP-2 mAb on wound healing, to enhance osseointegration of implants. The titanium beads utilized in the present study were relatively smooth. It has been demonstrated that titanium surface microtexture can significantly affect the binding and behavior of osteogenic progenitor cells [28–30]. Though rough surface of implants is initially conducive to greater degree and more accelerated osseointegration, these surfaces have the drawback of promoting biofilm attachment and possibly peri-implantitis. Immobilized anti-BMP-2 may be used as an alternative surface modification strategy to enhance osseointegration. Titanium granules can be considered as potential graft material for the repair of skeletal defects [31]. However, in some applications, it may be desirable to have a biodegradable scaffold, so that the regenerated tissue does not contain remnants of the scaffold material. Alginate hydrogel, a natural heteropolysaccharide, can be formulated as an injectable and biodegradable scaffold [32, 33] and has been used extensively in bone tissue engineering [17, 23]. Immobilization of anti-BMP-2 mAb on alginate scaffold will improve its bone regenerative properties. In such situations, alginate and collagen may be more appropriate options. There are many strategies available to modulate the rate of degradation of collagen by cross-linking [34] and alginate by oxidation [33]. Currently, we are investigating the physical properties of sites regenerated with each of these scaffolds using AMOR to characterize the physical strength of the regenerated tissues (manuscript in preparation). This information will further aid in the selection of appropriate scaffold for each tissue engineering application.

Taking into account the high degree of homology between BMP-2 and other osteogenic BMPs, such as BMP-4 and BMP-7, the binding capacity of murine anti-BMP-2 mAb with BMP-4 and BMP-7 has been examined* in vitro* and* in vivo *[16]. The cross-reactivity of murine anti-BMP-2 observed in our previous studies with BMP-4 and BMP-7 using ACS suggests that anti-BMP-2 immobilized on Ti and alginate might be able to capture multiple endogenous osteogenic BMPs, leading to* de novo* bone formation. This implies that the efficacy of AMOR may be in part attributable to the capacity of anti-BMP-2 mAb to capture multiple osteogenic mediators. In view of the significant degree of homology (92.2%) between the human and rat for BMP-2 proteins [16], the results of our calvarial defect model are likely to extend to clinical and translational applications of anti-BMP-2 mAb for mediating* de novo* bone regeneration. The feasibility of immobilizing this mAb on different types of scaffolds with unique physical properties makes this novel treatment modality even more versatile.

## 5. Conclusions

We report here on the application of immobilized murine anti-BMP-2 mAb to three different types of biomaterial to investigate their ability to mediate AMOR. The results demonstrated significant* de novo* bone formation with all three scaffolds immobilized with murine anti-BMP-2 mAb. Osseous defects regenerated with anti-BMP-2 mAb immobilized on collagen sponge and alginate exhibited more volumetric shrinkage than titanium. During early healing, significant cellular infiltration and adhesion were observed on scaffolds immobilized with murine anti-BMP-2 mAb. The present study demonstrated the possibility of utilizing different scaffolds with varying physical properties as scaffolds immobilized with anti-BMP-2 to participate in AMOR. These data have potential implications for the mechanism of action of AMOR, suggesting that anti-BMP-2 may capture endogenous osteogenic BMPs, which may in turn mediate* de novo* bone formation.
